# Structural analysis of a replication protein encoded by a plasmid isolated from a multiple sclerosis patient

**DOI:** 10.1107/S2059798319003991

**Published:** 2019-04-29

**Authors:** Turgay Kilic, Alexander N. Popov, Amelie Burk-Körner, Anna Koromyslova, Harald zur Hausen, Timo Bund, Grant S. Hansman

**Affiliations:** a Schaller Research Group at the University of Heidelberg, Heidelberg, Germany; b Department of Infectious Diseases, Virology, University of Heidelberg, Heidelberg, Germany; c Structural Biology Group, European Synchrotron Radiation Facility (ESRF), Grenoble, France; d German Cancer Research Center (DKFZ), Heidelberg, Germany; e Biosciences Faculty, University of Heidelberg, Heidelberg, Germany

**Keywords:** bovine meat and milk factors, episomal circular DNA, *trans*-acting replication initiator protein, multiple sclerosis, X-ray crystal structure

## Abstract

Bovine meat and milk factors (BMMFs) are circular, single-stranded episomal DNAs that have been detected in bovine meat and milk products. BMMFs are thought to have roles in human malignant and degenerative diseases. BMMFs encode a replication initiator protein (Rep) that is actively transcribed and translated in human cells. In this study, a Rep WH1 domain encoded on a BMMF (MSBI1.176) isolated from a multiple sclerosis human brain sample was determined to 1.53 Å resolution using X-ray crystallography. The overall structure of the MSBI1.176 WH1 domain was remarkably similar to other Rep structures, despite having a low (28%) amino-acid sequence identity. The MSBI1.176 WH1 domain contained elements common to other Reps, including five α-helices, five β-strands and a hydrophobic pocket. These new findings suggest that the MSBI1.176 Rep might have comparable roles and functions to other known Reps of different origins.

## Abstract 2

The crystal structure of the WH1 domain of the eukaryotic replication initiator protein (Rep) MSBI1.176 in the dimeric form has been determined to 1.53 Å resolution and shows a number of structural similarities to and differences from other known prokaryotic Reps.

## 1. Introduction   

The consumption of bovine meat and milk is considered to be a risk factor for the development of colon and breast cancers (Chan *et al.*, 2011 ▸; Corpet, 2011 ▸; Huxley *et al.*, 2009 ▸). Indeed, epidemiologic data suggest that there is a correlation of these cancers with the consumption of bovine products from cattle derived from Eurasian aurochs (zur Hausen & de Villiers, 2015 ▸; zur Hausen *et al.*, 2017 ▸; zur Hausen, 2012 ▸). Recently, it was suggested that bovine meat and milk factors (BMMFs), which are circular, single-stranded episomal DNAs (<3 kb) that are found in bovine meat and milk products, might represent a possible etiological agent of such diseases (Funk *et al.*, 2014 ▸; Falida *et al.*, 2017 ▸; zur Hausen *et al.*, 2019 ▸). More recently, BMMFs were isolated from patients with multiple sclerosis and studies suggested these to be a possible infectious agent of this disease (Whitley *et al.*, 2014 ▸; Lamberto *et al.*, 2014 ▸; Gunst *et al.*, 2014 ▸; zur Hausen *et al.*, 2019 ▸).

Typically, BMMFs encode an autonomous plasmid *trans*-acting replication initiator protein (Rep). Rep binds at an origin of replication on the DNA (termed *ori*) and in most cases comprises a set of repetitive DNA elements (termed iterons), which are present within most BMMFs (zur Hausen *et al.*, 2017 ▸). Replication of various plasmids, including circular Rep-encoding single-stranded (CRESS) DNA viruses, also requires the binding of the Rep to a specific DNA sequence (Kornberg & Baker, 1992 ▸). Within prokaryotes, Rep plays a central role in maintaining the plasmid copy number, as reported for the F plasmid in *Escherichia coli* (Kline, 1985 ▸). This regulation is also critical for the replication of plasmid-derived, bacteriophage-like or virus-like DNA genomes (Ruiz-Masó *et al.*, 2015 ▸). Reps are essential for the replication of multidrug-resistant bacteria in humans (Schumacher *et al.*, 2014 ▸) and studies have suggested that Reps have a role in transmissible amyloid proteinopathy (Molina-García *et al.*, 2018 ▸; Giraldo *et al.*, 2016 ▸, 2011 ▸).

Recently, an episomal circular DNA (isolate MSBI1.176, accession LK931491.1) was isolated from a brain sample of a patient with multiple sclerosis (Whitley *et al.*, 2014 ▸). The MSBI1.176-encoded Rep exhibits 98% amino-acid sequence identity to a Rep encoded on the Sphinx-1.76 genome (GenBank ADR65123.1 and HQ444404.1), which was isolated from culture and brain preparations of transmissible encephalopathy-related agents (Manuelidis, 2011 ▸). Moreover, there were indications of the detection of Sphinx-1.76-encoded Reps in neural cells (GT1 cell line) and brain samples of mouse CNS, hamster CNS and human glioblastoma based on Sphinx-1.76-correlated antibodies (Yeh *et al.*, 2017 ▸). Serology based on the MSBI1.176 Rep antigen showed positive immune responses for healthy human blood donors and indicated a possible pre-exposure towards these agents (Eilebrecht *et al.*, 2018 ▸). Therefore, deciphering the functions of BMMFs in human malignant and degenerative disease is becoming increasingly important.

The X-ray crystal structures of Reps have been well documented and the structural basis for autonomous replication has been described (Giraldo *et al.*, 2003 ▸; Komori *et al.*, 1999 ▸; Nakamura *et al.*, 2007 ▸; Swan *et al.*, 2006 ▸). Reps are composed of two winged-helix domains (termed WH1 and WH2) that are essentially a fused N- and C-terminal protein. Reps transform between monomeric and dimeric forms depending on their specific function and binding to DNA (see Forest & Filutowicz, 2003 ▸). Large structural changes involving both domains complement these oligomeric forms. The structural transformation requires certain α-helices and β-strands on the Rep to be refolded and/or shifted (Nakamura *et al.*, 2007 ▸). In the dimeric form, the Rep functions as a repressor, where WH2 binds to each operator DNA repeat and WH1 functions to form the dimerization interface. In the monomeric form, the Rep functions as a replication initiator, where WH1 undergoes a large structural movement, *i.e.* dimer dissociation, thereby allowing WH1 to bind to the iteron end, while WH2 binds to the opposite iteron end.

In this study, we determined the X-ray crystal structure of MSBI1.176 WH1 in the dimeric form to 1.53 Å resolution. Overall, the structures of MSBI1.176 WH1 and other Reps were remarkably similar, despite having low amino-acid sequence identities. Although structural differences were also observed, our findings suggested that the MSBI1.176 Rep might have similar roles and functions to other Reps. Moreover, this new structural information could be important for defining vulnerable regions on the Rep and possibly aid in future inhibitor design.

## 2. Materials and methods   

### 2.1. Protein expression and purification   

The MSBI1.176 DNA (LK931491.1) was isolated from a brain sample of a patient with multiple sclerosis (Whitley *et al.*, 2014 ▸). The MSBI1.176 WH1 domain (residues 1–135) was expressed in *E. coli* and purified as previously described for human norovirus protruding domains (Hansman *et al.*, 2011 ▸). Briefly, the codon-optimized WH1 was cloned in a modified pMal-c2X expression vector (GeneArt) and transformed into *E. coli* BL21 cells for protein expression. Transformed cells were grown in LB medium supplemented with 100 µg ml^−1^ ampicillin for 4 h at 37°C. Expression was induced with 0.75 m*M* IPTG at an OD_600_ of 0.7 for 18 h at 22°C. The cells were harvested by centrifugation at 6000 rev min^−1^ for 15 min and were disrupted by sonication on ice. His-tagged MBSI1.176 WH protein was initially purified from an Ni column (Qiagen), dialyzed in gel-filtration buffer (GFB; 25 m*M* Tris–HCl pH 7.6, 300 m*M* NaCl) with 10 m*M* imidazole and digested with HRV-3C protease (Novagen) overnight at 4°C. The cleaved MSBI1.176 WHI domain was then applied onto the Ni column again to separate and collect the cleaved protein, and dialyzed in GFB overnight at 4°C. The MSBI1.176 WH1 protein was further purified by size-exclusion chromatography, concentrated to 5 mg ml^−1^ and stored in GFB at 4°C.

### 2.2. Crystallization   

Crystals of MSBI1.176 WH1 grew using the hanging-drop vapor-diffusion method at 18°C in ∼6–10 days in a 1:1 mixture of protein sample and mother liquor (0.2 *M* magnesium acetate, 20% PEG 3350). Prior to data collection, MSBI1.176 WH1 crystals were transferred to a cryoprotectant containing the mother liquor with 40% PEG 3350, followed by flash-cooling in liquid nitrogen.

### 2.3. Data collection and processing, structure determination and refinement   

X-ray diffraction data for the MSBI1.176 WH1 domain were collected on beamlines ID23-1 and ID30B at the European Synchrotron Radiation Facility (ESRF). For the single-wavelength anomalous diffraction using native sulfur (S-SAD) experiments, diffraction data were collected from seven crystals at λ = 1.850 Å on beamline ID23-1 equipped with a Dectris PILATUS 6M pixel-array detector. The X-ray beam size at the sample position was 50 µm and the dimensions of the crystals were approximately 70 × 70 × 200 µm. To decrease the radiation-damage effects, the helical data-collection strategy was applied. One native data set was collected on ID23-1 at λ = 0.972 Å for initial phase extension and a second native data set was collected on ID30B at λ = 0.979 Å for structure refinement. Optimal experimental parameters for data collection were designed using *BEST* (Bourenkov & Popov, 2010 ▸) incorporated into the *MXCuBE* software (Gabadinho *et al.*, 2010 ▸) at the ESRF.

The single native data set was processed with *XDS*, while the multiple data sets for S-SAD were processed with *XDS* and then merged using *XSCALE* (Kabsch, 2010 ▸). Our initial attempts to solve the structure of MSBI1.176 WH1 by molecular replacement using prokaryotic RepA proteins as search models failed. Therefore, several data sets were collected for further processing using S-SAD (Liu *et al.*, 2012 ▸). The S-SAD phasing protocol was carried out using the *SHELXC*/*D*/*E* pipeline as implemented in *HKL*2*MAP* (Sheldrick, 2010 ▸). 1000 trials were carried out for substructure determination in *SHELXC*. Using a resolution of 2.3 Å and an anomalous signal truncated to 3.1 Å, where the self-correlation coefficient for the anomalous signal decreased to 25%, *SHELXD* correctly identified all 24 sulfur sites. 415 residues were built automatically by *SHELXE*, which resulted in an interpretable map for further processing. Finally, *ARP*/*wARP* was used for automated model building based on the first native data set collected (Langer *et al.*, 2008 ▸). The structure was refined using the second high-resolution data set in multiple rounds of manual model building in *Coot* (Emsley *et al.*, 2010 ▸) and *PHENIX* (Adams *et al.*, 2010 ▸). The structure was validated using *MolProbity* and *PROCHECK*. Interactions were analyzed using *Accelrys Discovery Studio* (v.4.1), with hydrogen-bond distances of between 2.4 and 3.5 Å. Figures and protein contact potentials were generated using *PyMOL*. Atomic coordinates and structure factors have been deposited in the Protein Data Bank (PDB) with accession code 6h24.

## 3. Results   

### 3.1. X-ray crystal structure of MSBI1.176 WH1   

**Figure 1:**
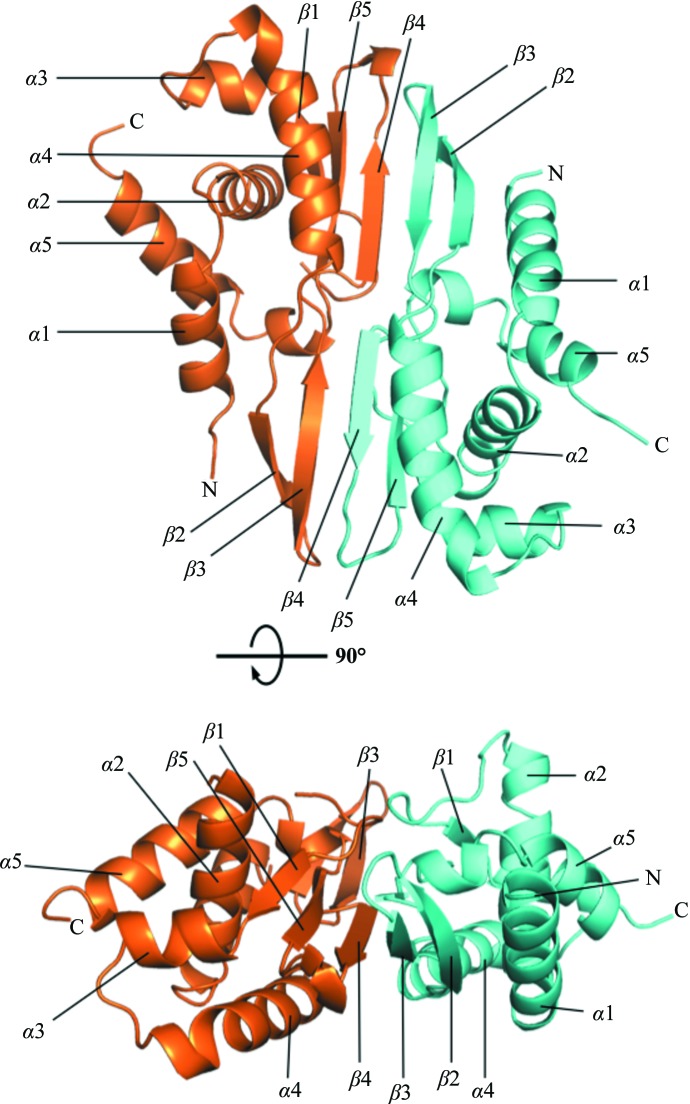
The X-ray crystal structure of the MSBI1.176 WH1 dimer. The MSBI1.176 WH1 protomers are colored cyan for chain *A* and orange for chain *B*. One protomer comprises five α-helices (α1–α5) and five β-­strands (β1–β5). The dimeric interface involves two β-strands (β4–β3).

The structure of MSBI1.176 WH1 (residues 2–133) was solved to 1.53 Å resolution (data statistics are given in Table 1 ▸). The asymmetric unit consisted of one WH1 dimer, *i.e.* two protomers (termed *A* and *B*). The electron density was well resolved for most of the protein (average *B* factor of 29.98 Å^2^). However, residues 36–39 could not be fitted into the *B* protomer owing to a lack of discernible electron density, although the electron density was distinct in the other protomer. The WH1 structure comprised five α-helices (α1–α5) and five β-strands (β1–β5) in each protomer (Fig. 1 ▸). The *A* and *B* protomers were closely related (r.m.s.d. of 0.37 Å); however, a minor structural shift was observed at the β2–β3 hairpin, suggesting some flexibility of this region. Importantly, with the improved resolution over those of previous structures (Giraldo *et al.*, 2003 ▸; Komori *et al.*, 1999 ▸; Nakamura *et al.*, 2007 ▸; Swan *et al.*, 2006 ▸), water molecules were effectively added to this Rep structure.

### 3.2. Structural comparison with other replication proteins (Reps)   

**Figure 2:**
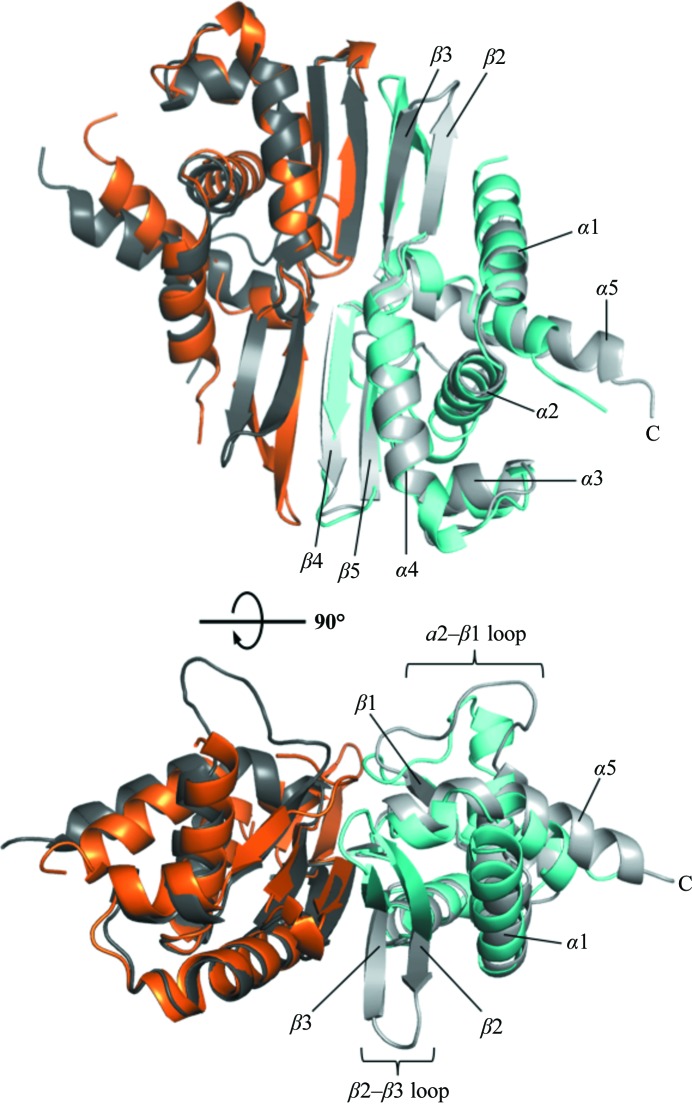
Structural comparison with the closely matching prokaryotic RepA WH1. MSBI1.176 WH1 and RepA WH1 have 28% amino-acid identity. Superposition with RepA (gray) showed that these two WH1 dimers are highly similar, with an r.m.s.d. of 1.20 Å. Structural differences in extended loops were observed, noticeably the loops connecting α2 and β1 as well as β2 and β3.

A database search for closely related structures and sequences revealed that MSBI1.176 WH1 has 28% and 17% amino-acid identity to *Pseudomonas syringae* RepA WH1 (RepA; PDB entry 1hkq; Giraldo *et al.*, 2003 ▸) and *E. coli* RepE (PDB entry 1rep; Komori *et al.*, 1999 ▸), respectively. Similar to that from RepA, the MSBI1.176 WH1 was also folded as the replication-inert dimer, while RepE (a WH1–2 construct) was crystallized in the monomeric initiator form. Superposition of MSBI1.176 WH1 and RepA WH1 showed that these two domains were structurally similar (r.m.s.d. of 1.20 Å), with both having the typical five α-helices and five β-strands (Fig. 2 ▸). A number of structural similarities and differences were observed between these two Reps.

**Figure 3:**
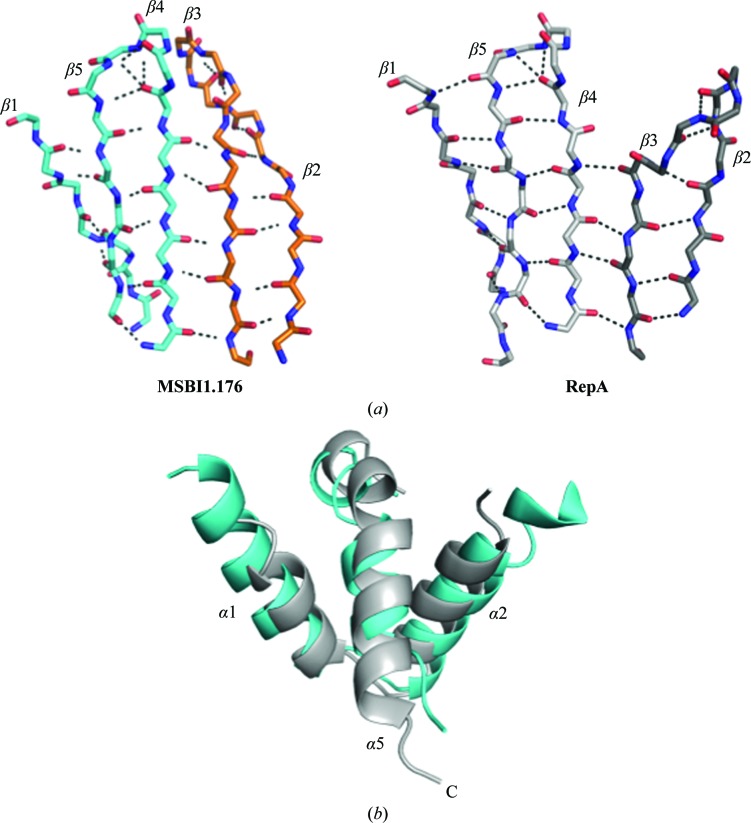
Structural similarities of MSBI1.176 WH1. (*a*) The five β-sheets (β1–β5–β4–β3–β2) showing the main-chain interactions in MSBI1.176 WH1 (cyan and orange) and RepA WH1 (gray). The β-­strands were held by numerous main-chain hydrogen bonds (dashed lines), similar to RepA, including the dimeric interface (β4–β3). (*b*) The region containing α1–α2–α5 was similar in orientation to that in RepA. This region produced a V-shaped structure and α5 is the linker region to the WH2 domain. The hydrophobic pocket also contained three leucine residues, *i.e.* Leu11, Leu18 and Ile25, which were similarly positioned in RepA (data not shown).

The dimeric interface of MSBI1.176 and RepA, which involves β3–β4, was held with a similar number of main-chain binding interactions, although not at identical residues (Fig. 3 ▸ *a*). This result suggested that the dimeric interface feature was likely to be related to function among the diverse Rep isolates. We also observed that water molecules bound at this dimeric interface (data not shown). However, how these water molecules stabilize the dimeric interface and/or are displaced after binding DNA and changing conformation is not yet known.

We also observed that the MSBI1.176 WH1 region that comprised α1–α2–α5 was similar in orientation to that in RepA, having the typical α1–α2 bend and thereby making a V-shaped structure (Fig. 3 ▸ *b*). This region, which forms the linker to WH2, also contains the hydrophobic heptad pocket, which typically contains a number of leucine residues (for example Leu12, Leu19 and Leu26 in RepA, and Leu24, Leu31 and Leu39 in RepE). The MSBI1.176 WH1 hydrophobic pocket also contained three leucine residues, *i.e.* Leu11, Leu18 and Ile25, which were similarly positioned as in RepA. Not surprisingly, water molecules were absent in the hydrophobic pocket of MSBI1.176 WH1.

**Figure 4:**
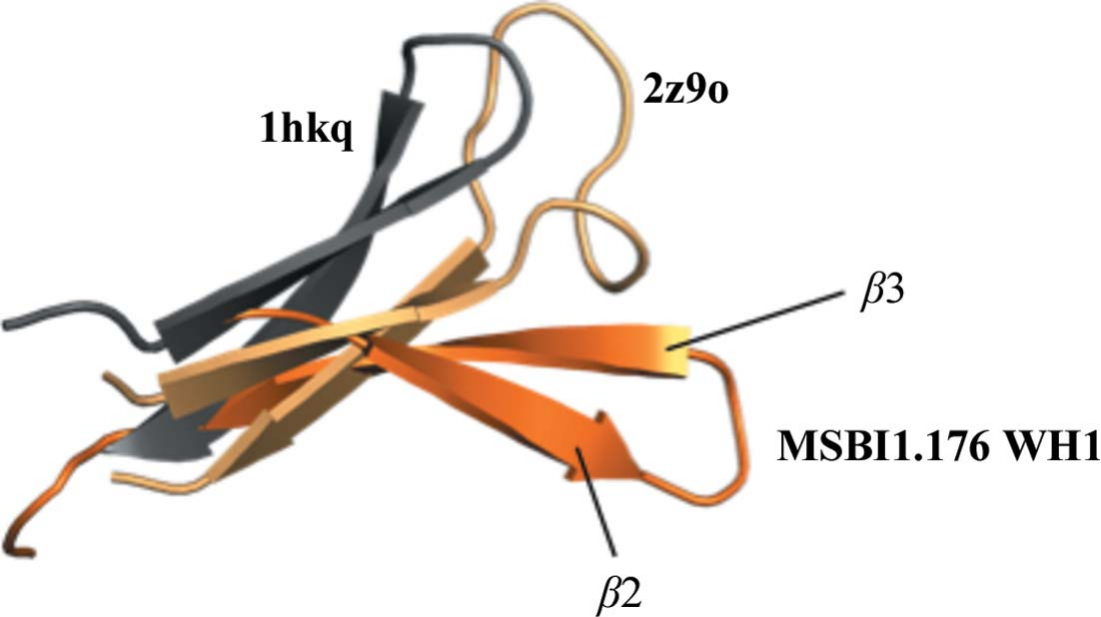
Superimposition of the MSBI1.176 WH1 β2–β3 hairpin onto prokaryotic Rep proteins with PDB codes 1hkq (RepA) and 2z9o (RepE). The MSBI1.176 WH1 β2–β3 hairpin shifted approximately 23 Å when compared with the equivalent RepA hairpin. The MSBI1.176 WH1 β2–β3 hairpin was held by direct main-chain interactions (Fig. 3 ▸ *a*) as well as water-mediated interactions. Note the different β2–β3 hairpin twists between the MSBI1.176 and RepA WH1 structures in Fig. 3 ▸(*a*). The RepE β2–β3 hairpin was positioned between these two WH1 β2–β3 hairpins.

In general, many of the structural features of MSBI1.176 WH1 are conserved in other known dimeric Rep structures (Giraldo *et al.*, 2003 ▸; Komori *et al.*, 1999 ▸; Nakamura *et al.*, 2007 ▸; Swan *et al.*, 2006 ▸). However, loop movements and different α-helices and β-strands have been observed among the different structures. In the case of MSBI1.176, the β2–β3 hairpin shifted approximately 23 Å when compared with the RepA β2–β3 hairpin (Fig. 4 ▸). In the case of RepE, the equivalent β2–β3 hairpin (residues 97–110) was not added to the structure since electron density was lacking (Giraldo *et al.*, 2003 ▸, 2011 ▸). It was suggested that the RepE β2–β3 hairpin was flexible and this flexibility might function by destabilizing the antiparallel β2–β3 hairpin and blocking dimerization (Giraldo *et al.*, 2003 ▸, 2011 ▸). However, the MSBI1.176 WH1 β2–β3 hairpin was clearly held by direct main-chain interactions, not unlike the RepA structure (Fig. 3 ▸ *a*). Moreover, we perceived that water-mediated interactions at this dimeric interface might also add further stability to this hairpin (Fig. 3 ▸ *b*).

### 3.3. Modeling of DNA binding   

**Figure 5:**
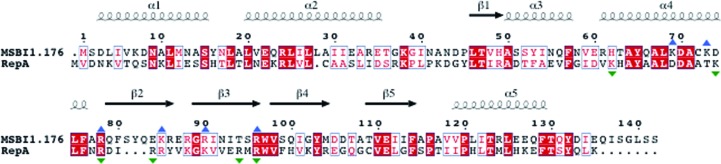
Amino-acid sequence alignment of MSBI1.176 (LK931491.1) and RepA (PDB entry 1hkq) using *ClustalW* (Genetyx); the figure was generated using *ESPript* (Robert & Gouet, 2014 ▸) with slight modifications. Secondary-structural elements are shown and were confirmed from the crystal structure. Identical and homologous residues are highlighted on a red background and as red letters, respectively. Presumably, a DNA molecule would interact along the dimeric interface and possibly with six basic residues in this region, *i.e.* Lys69 (α4), Lys73 (α4), Arg78, Lys85 (β2), Arg90 (β3) and Arg96. The basic amino-acid residues of RepA that are suggested to participate in DNA interaction are marked with green triangles and the equivalent residues in MSBI1.176 WH1 are marked with blue triangles.

Previous modeling analysis of the RepA domain indicated that six basic residues on α2, β2, β3 and adjacent loops (Lys74, Arg81, Arg91, Arg93, Lys62 and Arg78; RepA numbering) might follow the minor groove of a DNA backbone (Giraldo *et al.*, 2003 ▸). In the MSBI1.176 WH1 structure, six basic residues were also found in this region, *i.e.* Lys69, Lys73 (both located on α4), Lys85 (β2), Arg90 (β3), Arg78 and Arg96 (both on adjacent loops). Although the electron density for the Lys73 (chains *A* and *B*), Lys85 (chains *A* and *B*) and Arg90 (chain *A*) side chains in MSBI1.176 WH1 was weak, two of these residues (Arg78 and Arg96) were at equivalent positions in RepA and were suggested to interact with a DNA molecule (Giraldo *et al.*, 2003 ▸; Fig. 5 ▸). The function of the MSBI1.176 WH1 β2–β3 sheet orientation is not obvious, although MSBI1.176 WH1 has a three-amino-acid insertion in the β2 strand that extended the sheet. Presumably, this insertion elegantly shifted Lys85 (β2) and Arg90 (β3) on the β2–β3 sheet in MSBI1.176 WH1 when compared with the equivalent RepA residues Arg81 and Arg91. Seen in another way, the MSBI1.176 β2–β3 sheet was rather flattened, whereas the RepA β2–β3 hairpin was hooked in the opposite direction (Fig. 2 ▸).

## 4. Conclusions   

Reps are important for the replication of plasmids or autarkic episomal nucleic acids in different hosts. It is speculated that such proteins and Rep-encoding DNAs might be linked to disease. Thus, careful structural and functional characterization of Reps is needed. The Rep described in this study is encoded by the human bioactive bovine meat and milk factor MSBI1.176, which was isolated from a patient with multiple sclerosis (Whitley *et al.*, 2014 ▸). Rep-specific serum antibodies have been found in a set of healthy human blood-bank donors, indicating general human exposure to such agents (Eile­brecht *et al.*, 2018 ▸). The discovery that this MSBI1.176-encoded Rep WH1 protein was closely similar to a prokaryote Rep structure might have important consequences and point towards a possible disease-correlated adaptation of these agents towards humans. This new structural information might aid in the development and design of therapeutic/preventive drugs that can inhibit these Reps of diverse origin.

## Supplementary Material

PDB reference: MSBI1.176 WH1 domain, 6h24

## Figures and Tables

**Table 1:** Data-collection and refinement statistics for the MSBI1.176 WH1 protein structure Values in parentheses are for the highest resolution shell.

	S-SAD	S-SAD, native	Native
Data collection			
ESRF beamline	ID23-1	ID23-1	ID30B
Wavelength (Å)	1.850	0.972	0.979
No. of crystals	7	1	1
Space group	*P*2_1_	*P*2_1_	*P*2_1_
*a*, *b*, *c* (Å)	104.86, 43.96, 107.71	104.86, 43.96, 107.71	32.38, 77.77, 47.68
α, β, γ (°)	90, 97.72, 90	90, 97.72, 90	90, 90.66, 90
Resolution range (Å)	19.91–2.30 (2.38–2.30)	19.91–1.58 (1.63–1.58)	40.65–1.53 (1.58–1.53)
*R*_merge_ (%)	11.20 (52.00)	4.00 (92.70)	4.09 (68.48)
*R*_meas_ (%)	11.70 (60.20)	4.40 (101.10)	4.45 (74.87)
CC_1/2_ (%)	100.00 (98.10)	99.90 (51.40)	100.00 (85.00)
〈*I*/σ(*I*)〉	33.30 (9.60)	15.80 (1.30)	20.91 (1.98)
Completeness (%)	99.90 (98.30)	98.30 (95.3)	99.42 (96.93)
Multiplicity	84.0 (28.5)	3.7 (3.4)	6.4 (6.1)
Refinement			
Resolution range (Å)			40.65–1.53
No. of reflections			35692
*R*_work_/*R*_free_ (%)			18.55/21.16
No. of atoms			
Protein			2028
Water			152
Average *B* factors (Å^2^)			
Protein			29.98
Water			33.17
R.m.s.d.			
Bond lengths (Å)			0.006
Bond angles (°)			1.080
Ramachandran plot (%)			
Favored			99.61
Allowed			0.39
PDB code			6h24
